# The co-creation of meaningful action: bridging enaction and interactional sociology

**DOI:** 10.1098/rstb.2015.0378

**Published:** 2016-05-05

**Authors:** Hanne De Jaegher, Anssi Peräkylä, Melisa Stevanovic

**Affiliations:** 1 Department of Logic and Philosophy of Science, IAS-Research Centre for Life, Mind, and Society, University of the Basque Country, San Sebastián, Spain; 2 Department of Informatics, Centre for Computational Neuroscience and Robotics, and Centre for Research in Cognitive Science, University of Sussex, Brighton, UK; 3 Department of Social Research, Finnish Center of Excellence on Intersubjectivity in Interaction, University of Helsinki, Helsinki, Finland

**Keywords:** social interaction, coordination, interactional autonomy, Goffman, conversation analysis, participatory sense-making

## Abstract

What makes possible the co-creation of meaningful action? In this paper, we go in search of an answer to this question by combining insights from interactional sociology and enaction. Both research schools investigate social interactions as such, and conceptualize their organization in terms of autonomy. We ask what it could mean for an interaction to be autonomous, and discuss the structures and processes that contribute to and are maintained in the so-called interaction order. We also discuss the role played by individual vulnerability as well as the vulnerability of social interaction processes in the co-creation of meaningful action. Finally, we outline some implications of this interdisciplinary fraternization for the empirical study of social understanding, in particular in social neuroscience and psychology, pointing out the need for studies based on dynamic systems approaches on origins and references of coordination, and experimental designs to help understand human co-presence.

## 1. Introduction

Imagine meeting someone you know has the same cultural background as you do, somewhere abroad, where the convention of greeting is different from what you share with this person. How will you greet each other? Will you offer your cheek? Or move in for a hug? How will you make sure you do not end up kissing on the lips? It may be a little awkward, but you will eventually end up greeting each other. You will coordinate and negotiate a social event. In so doing, you will co-create a meaningful action, which neither of you could have done alone, or outside of its particular context.^1^

Three mutually influencing systemic levels are at stake here: (i) single individuals who, (ii) in a particular societal and cultural context, come together in (iii) a face-to-face interaction.^2^ Here, we focus on what happens at the intersection of these three levels in order to better understand the co-creation of meaningful action.

The co-creation of action or meaning—part of intersubjectivity described in its broadest sense as social understanding—has fallen somewhat into the cracks between mainstream sociology and cognitive science because, traditionally (and putting it bluntly), the one has mainly been interested in socio-cultural norms and organization, and the other mainly in individual cognition. Two elements have largely been missing from both fields. Firstly, face-to-face interactions—the central and primary locus of the co-creation of meaningful action. And secondly, action and meaning, in both fields, have been taken for granted, either as culturally given (e.g. shared norms), or determined by individual predispositions (e.g. in terms of internal representations). Therefore, the *generation* of *meaningful action* has fallen out of focus. There is no answer to the questions: What makes possible the co-creation of action and meaning? In which ways do the interplays (including tensions) between face-to-face interactions and the individual, and between face-to-face interactions and the historico-socio-cultural context contribute to that process?

Two approaches, one within sociology and one within cognitive science, seem well poised to address these questions: interactional sociology, and the enactive approach to intersubjectivity. The first approach was started by Erving Goffman (e.g. [1,2]) whose ethnographic studies described the organization of face-to-face interaction. Goffman's research questions were taken up by conversation analysts (e.g. [3,4]) who developed a method to investigate audio- and video-recordings of naturally occurring interactions, to reveal the basic structural organizations that make ordered interaction possible. The second approach, enaction, is a naturalistic, non-reductionist approach to cognition defined as sense-making, which is the relational process of signification between an autonomous, self-organizing, embodied, animate agent and the world. The agent's perspective on and understanding of their world is based in their self-organization, because it entails certain needs and constraints [5,6]. Intersubjectivity, then, is the way in which lived, situated, bodily coordinations between such cognizers form and transform the ways in which they make sense of each other and the world together [7].

Both approaches seek to overcome blindnesses to the interactional generation of meaningful action of their respective mother disciplines (sociology and cognitive science). We believe both can contribute to answering the question—interactional sociology for its careful scrutiny of organizational properties of interactions; and enaction for its definition of both social interaction processes and of individuals in terms of autonomy, and for its study of their interplay in terms of participatory sense-making [7].

In this paper, we bring these fields into a dialogue. Together they can elucidate the question of what enables the co-creation of meaning. We first investigate, in §2, face-to-face interactions and analyse their organization in terms of the autonomy of interaction processes and the elements that sustain their organization. In §3, we delve deeper into the interplay between this interactional self-organization and individual self-organization, since it is in this interplay that actions and meanings are collaboratively created. Throughout the paper, we explicate and interweave terms, conceptualizations and examples from interactional sociology and enaction. This braiding of the approaches allows us, in §4, to propose ways in which social interaction's role in the co-creation of meaning can be empirically researched, particularly in social neuroscience and in psychology.

## 2. The organization of interaction

To understand the co-creation of action or meaning, we need to get a firm grip on the organization of social interaction. Possibly one of the most fruitful ways to approach interactional organization—one that is unique to both fields, and sets them apart from other disciplines—is to view the interaction process as an autonomous system.

When looking for an account of interaction as an autonomous system, we are taking up a perspective that in recent decades has been conceptualized as relational sociology: a perspective on social life that foregrounds unfolding relations rather than individual or collective substances [8]. At the level of macro-sociology, in considering ‘how resources, goods, and even positions flow through particular figurations of social ties’ ([8], p. 298), this perspective has yielded the social-network analysis of White (e.g. [9]) and many others. In this paper, however, we focus on the micro-sociology of such relations as they appear in face-to-face social interactions. Here, Erving Goffman can be considered as the most influential figure. He has most convincingly formulated the idea of autonomy of social interaction. For him, the autonomous realm of the ‘interaction order’ should be treated ‘as a substantive domain in its own right’, in which ‘the contained elements fit together more closely than with elements beyond the order’ ([2], p. 2).

But what does it mean for the elements to ‘fit together more closely’? Goffman never defined it, but enaction does. For enaction, a system—be it a social interaction or a living system—is autonomous when it is composed of processes that actively generate and sustain an identity under precarious conditions [10,11]. An autonomous system self-organizes, and hence forms an identity and differentiates itself from the environment. To generate an identity means to possess the property of operational closure. This means that for any constituent process of the system, there are always one or more other processes in the system that enable it or are a condition for it (i.e. there are no processes that are not conditioned by other processes in the network, though conditions external to the system may also be necessary for any of the system's processes). The conditions under which the system self-organizes are *precarious*, which means that if the system was not organized like a network of processes, under otherwise equal physical conditions, isolated component processes of the system would tend to run down or extinguish. In other words, an autonomous system depends for its organization and self-maintenance on its component processes and their relations, and they in turn depend on the network. Autonomy defined in this way has been proposed to emerge and to generate identities at different levels, ranging from metabolic, over sensorimotor, neuro-dynamical, immune and social systems [6,10–14]. The fact that such a system is precarious, moreover, means that it may not be able to deal with all the perturbations that can happen to it. We will pick up the theme of precariousness again in §3.

What can this enactive definition of autonomous systems mean for exploring the Goffmanian interaction order? First, enaction proposes that an autonomous system differentiates itself from the environment, forming an operational closure. In interactional sociology, the environment from which the interaction order differentiates itself has been understood as consisting of two other organizations: (i) large-scale social institutions, and (ii) individual actors. This means that the ‘contained elements’ of the interaction order (i.e. structures and practices that organize situated social interactions; see below) are to be distinguished from these two organizations.

The large-scale social institutions from which the interaction order differentiates itself include durable social structures such as legal or economic orders, family relations or, more generally, cultural orientations and expectations. The face-to-face social interaction order, albeit linked to these socio-cultural structures, has its own properties that are not defined by them. This shows in the fact that the key rules and practices of social interaction remain there, regardless of the socio-cultural environment of the interaction. How these practices are realized is informed by the socio-cultural contexts [15], but recognizably same practices can be found across those contexts: as Goffman puts it, ‘pedestrian traffic rules can be studied in crowded kitchens as well as crowded streets, interruption rights at breakfast as well as in courtrooms, endearment vocatives in supermarkets as well as in the bedroom’ ([2], p. 2).

The other organization from which the interaction order distinguishes itself consists of the individuals participating in interaction. The operational closure of the interaction shows up in the way in which the rules and practices of social interaction are ‘social facts’: highly conventionalized and rooted in strong social norms. While all their realizations take place through individuals' actions that require cognitive, emotional, perceptual and attentional competences, the rules and practices themselves have persistence over any single acting individual and individual differences (e.g. [4,16]). In social encounters, they both enable and constrain the participating individuals' actions, thereby generating the identity of the interaction as a self-organized system, which cannot be reduced to things like individual actors' communicative intentions.

While interaction as an autonomous system *differentiates* itself from the environment, it does not *isolate* itself. In the enactivist view, processes within the operational closure of an autonomous system can be linked to processes external to the system, and conditions external to the system may well also be necessary for any ‘within-system’ processes. This resonates with Goffman [2], who, in discussing the relationship between the interaction order and the other social orders, suggested that ‘[e]xploring relations between orders is critical, a subject matter in its own right’ ([2], p. 2). As for the linkages between interaction as an autonomous system, and the acting individuals, studies on how conversations reshape participants' memories [17] are a case in point, showing that the organization of conversation, in terms of its constellation of social roles, is consequential upon the ways in which the participants' memories are reformed. In §3, we will specifically go into the question of how the individual as a system relates to the systemic properties of social interaction.

Let us now turn to the very organization of interaction as an autonomous system. What are the key features of this organization, and how do they relate? Both interactional sociology and enaction view the autonomy of social interaction as arising from the coordination of behaviours. A basic difference between the two fields is that interactional sociology approaches the coordination of behaviours in a more structural way—speaking of structures and practices as normative principles that are there, as social facts, prior to any situated social interaction, while enaction views coordination more in terms of emergent processes. This different orientation stems from differences in background and methodology. It is not a very strict division, and each field also has affinities with the other's perspective. Initially, however, interactional sociology and enactivism differ in terms of their perspectives, prioritizing either structure or process. Taking this as a basis for discussion is handy for illuminating how both fields can mutually inform and enhance each other.

We discuss coordination of behaviour in interaction first from a more structural perspective, and then from a more processual perspective, in each case also already indicating and searching overlaps and connections between the two starting points, as well as drawing attention to aspects of the autonomy of interaction.

### (a) Structural perspective on coordination

In this section, we extract from contributions by Goffman and conversation analysts four interlocking domains of coordination: *co-presence*, *engagement*, *turn-taking* and *sequentiality*. Each of these has identifiable structures that organize interaction.

#### (i) Co-presence

Goffman defined social interaction as what happens in co-presence: as ‘that which uniquely transpires in (…) environments in which one or more individuals are physically in one another's response presence’ ([2], p. 3). The co-presence involves ‘mutual monitoring possibilities’ where the participants are accessible to each other's senses [18,19]; it is the most primordial domain of coordination with identifiable structural features.

Co-presence brings along both strong normative orientations, i.e. what ought and ought not to be done in the others' presence, and a special experiential state, i.e. awareness of the others' presence. Even when the co-present participants are ‘just’ in the same place at the same time, without being directly involved in joint actions such as conversation, they will still strongly orient themselves to the others' presence, by attending to cultural rules that ‘establish how individuals are to conduct themselves by virtue of being in a gathering’ ([18], p. 135). Such rules define, for example, the main involvement in each type of gathering (e.g. exercising in a gym, reading and writing in a library), how additional involvements are to be organized vis-à-vis the main one, as well as the expected physical appearance and patterns of movement.

Individuals are virtually unable to ignore co-presence and the rules arising from it, at least not without a considerable moral cost. Co-presence stands out as something that the individuals have to take into account in all their actions. Being in an elevator with another person, while acknowledging their presence without initiating a closer encounter, is a situation where the structural features of co-presence manifest themselves in a condensed form: in all our doings, we are directed to the others' body postures, gaze directions, movements, them perceiving us and being perceived by us. In all forms of co-presence, we exercise similar, albeit less conscious attention to the others' presence. What emerges out of such situations is never under the control of any single individual. Even trying *not* to have an encounter is a coordinated achievement of several participants producing a behavioural pattern with certain self-organizing properties.

#### (ii) Engagement

The other key domain of coordination has to do with people's management of the cognitive, affective and behavioural involvement in the interaction. There are two basic forms of co-presence that Goffman distinguished throughout his career: *gathering* and *encounter* (e.g. [18,19]). A gathering involves ‘mere’ co-presence, attendance to the cultural rules briefly discussed above. An encounter, then again, involves a new layer of engagement: Goffman characterized the encounter by saying that its participants ‘jointly ratify one another as authorized co-sustainers of a single, albeit moving, focus of visual and cognitive attention’ ([19], p. 134). The participants attend to each other, what they refer to, or observe together in the environment.

The autonomy of social interaction vis-à-vis its individual participants is manifested in the engagement of the participants in their shared focus of attention. The participants' behaviours become coordinated through this mutual immersion in the interaction. In spite of its apparent spontaneity, such engagement has strong socio-normative backing: it is expected from the participants of an encounter ([19], p. 134) and, accordingly, the initiations and terminations of encounters are surrounded by rituals that facilitate coordination at the critical junctures between encounters, where such an expectation holds, and gatherings, where such an expectation does not hold.

#### (iii) Turn-taking

The third domain of coordination in social interaction has to do with distribution of opportunities for talk. The distribution of turns at talk is based on rules that have durability over time and across individuals. These rules constrain the actions of the individual participants. Sacks *et al*. [3] outlined their key features. Here, the coordination of behaviour is based on the participants' incessant orientation to norms and conventions providing that: (i) Verbal communication in social encounters is organized in turns at talk that alternate between the participants. (ii) Current speaker is initially entitled to one turn constructional unit (smallest amount of talk that in its sequential context counts as a turn). (iii) The completion of such a unit is a transition relevance place where the speaker change may occur. (iv) A current speaker may select the next; if (s)he does not do that, any participant can self-select at the transition relevance place; and if even that does not happen, the current speaker may (but need not) continue.

Virtually all spoken utterances are produced and received in the structural matrix provided by turn-taking rules (cf. [20])—and this seems to hold for signed utterances as well [21]. Many institutional settings involve specific applications of these rules [15], but also in these settings, the coordination of behaviour is a result of all the participants abiding by the specific turn-taking rules characteristic of that particular institution.

#### (iv) Sequentiality

The fourth domain of coordination in social interaction involves the relationship between utterances, including non-verbal communicative actions, as they occur one after the other. Social norms and expectations tie consequent communicative actions together in specific, partially predictable ways. These conversational structures also establish interaction as autonomous vis-à-vis the individual actors: they enable and constrain all communicative action.

Single acts are parts of larger entities [4]. The most basic and the most important among such entities is what in conversation analysis is called adjacency pair [22]: a unit consisting of two actions in which the first action performed by one participant invites a particular type of second action to be performed by their co-participant. Typical examples of adjacency pairs include question–answer, greeting–greeting and request–grant/refusal. The first action generates a pressure towards the projective second speaker to produce a relevant response. Thereby, the adjacency pair structure enables the possible first speakers to elicit specific responses from others (by for example asking a question). At the same time, it is in and through their response, that also the second speaker has a possibility to define the status of the first speaker's behaviour as action (a question ‘is’ a question inasmuch as it is responded to with an answer). Hence, the autonomy of the interaction is revealed by the adjacency pairs being more than their constituent parts. Neither a first pair part nor a second pair part can be defined without the other, but they are part of one and the same process ([23], p. 286).

By now, we have discussed four domains of coordination of behaviour in interaction—co-presence, engagement, turn-taking and sequentiality—where normatively based structures exceeding time and space facilitate coordination, and thereby contribute to the autonomy of social interaction. These domains also hang together, conditioning each other. In social interaction, co-presence is the primary domain of coordination. When there is co-presence, engagement becomes possible (but engagement is not necessary for there to be co-presence). Engagement, then, is necessary for the remaining two domains of coordination, turn-taking and sequentiality—shared attention and involvement are needed for taking turns and for producing sequentially organized actions. Overall, these four domains of coordination are constituent parts of the kind of ‘participation structure’ ([24], p. 52) that characterizes all human social interaction (see also the first model in [25]).

### (b) Emergent processual perspective on coordination

Now, let us take a look at how enaction views the organization of interaction. Unlike interactional sociology, which highlights the structures that facilitate coordination, enaction describes interactional organization in terms of dynamic, emergent processes of coordination. Coordination is a ubiquitous phenomenon in and between biological systems [26,27], and much of the coordination that happens in social interactions does not require high-level cognitive skills [7]. Enaction analyses the complex temporality of coordination, different kinds and references of coordination and its relationship with social agency.

#### (i) The temporality of coordination

Coordination happens at multiple timescales [27], ranging from fast neural and physiological coordination, over mid-range behavioural and gestural coordination within single encounters, to the longer term continuity of interactional patterns and topics of talk over consequent encounters or, more broadly, interaction histories and inter-personal relationships.^3^ Enaction deals with this temporal complexity and multilayeredness by using the mathematical tools of dynamical systems theory (e.g. [28,29]).

Further aspects of interactional coordination's complex temporality are, for instance, degrees of coordination. Kelso describes how a child and an adult walking together (and having to adjust their strides to each other) do not mostly move in perfect synchrony (‘absolute’ coordination), but rather *relatively* coordinate, meaning that they move into and out of zones of high synchrony [26]. The coordination associated with interactional organizations such as turn-taking are also usually relative in this sense. The turn-taking system allows for different sizes of turn [3], and turns at talk are designed so that a transition space becomes recognizable for the participants. The next speaker typically starts their utterance at some point during this transition space but not exactly at a given point in time [20]. Coordination occurs in the fine tuning of speaker transitions, while there is variation in the ways in which this is temporally organized.

#### (ii) Origins and references of coordination

Coordination can also be of many kinds in terms of its origins or reference points [30,31]. We can conceptually distinguish between at least *external coordination*, *pre-coordination*, *functional coordination*, *coordination-with* and *coordination-to*. External coordination happens when several people are each coordinated to a third event or process, e.g. their aerobics teacher, or a film, and therefore seem to be coordinated with each other in moving in the same way, making similar expressions, or showing similar neural activations at the same time. People are pre-coordinated when they act together according to a shared history, cultural patterns, societal norms, roles or institutions, or even their personal shared interactional history (e.g. a national or a familial greeting custom). Functional coordination is coordination that serves (has a function for) the interaction, e.g. arranging to meet, i.e. to interact later. Finally, coordination-with and coordination-to mark the difference between a mutual or co-regulated coordination and an individual, singular attunement to a patterned event or process. For instance, I coordinate with you over a smoothly running video-connection, but if I were later to view a recording of what I saw you doing before, I can only coordinate *to* that. It is, in fact, hardly possible for humans (and animals) to purely ‘coordinate-to’ (and it has repeatedly been shown that attempts to coordinate-with when it is only possible to coordinate-to can be upsetting and disruptive of social understanding, see [32–34]). Coordination is also highly dependent on embodied and situated constraints that affect it, and which can and should be taken into account when investigating it.

To illustrate the robustness of coordination and the interplay of its structural and dynamical elements, we may think about telephone closings, where the turn-taking apparatus has to be stopped. For this purpose, people resort to specific closing rituals—i.e. social practices by which the pressure to keep coordinating can be relaxed. As shown by Schegloff & Sacks [22], the move to closing is often instigated through the exchange of ‘okays’, which is then followed by reciprocal salutations, well-wishes, saying each other's name, endearment terms and the like. Furthermore, such closings are usually rhythmically well integrated—i.e. there is an isochronous structure of prosodically prominent syllables, whose tempo normally accelerates so that the final salutations are either latched to each other or produced on the same rhythmic beat [35]. Telephone closings thus provide a clear example of how collaborative systemic dynamics and structural patterns may be intertwined in the unfolding of social interaction: the closing ritual has a wide normative backing but, each time, its realization is an emergent product of two participants' interactional contributions. Besides, the example is indicative of the autonomy of interaction: to break the expectations of continuity requires special effort and ritual care.

Let us come back to the example of greeting. The acquaintances meeting each other abroad orient themselves to conflicting sets of norms and conventions pre-coordinating the greeting ritual. There are their shared native cultural norms, but also those of the country they now are in. Furthermore, the participants have their relational history, involving prior greetings and shared memories of encounters with particular degrees of intimacy and deference. The greeting is therefore heavily pre-coordinated, but this alone does not suffice to organize it. Necessarily, the greeting participants will have to coordinate-with: one will notice the other first and will attract their attention; situational constraints permitting, they will approach each other; one will indicate first, with body movements, willingness to engage in a particular type of salutation (be it kiss, embrace, handshake or mere verbal salutation), and the other will either align with this, or engage in another type of salutation. This coordination-with may be mingled with elements of coordination-to; as Kendon [36] shows, initiating a greeting ritual involves one participant attracting the attention of the other: at the moment when I have noticed you but you have not yet noticed me, I am arguably coordinating my actions *to* you. We also find functional coordination: the greeting opens up a state of heightened mutual access between the participants ([37], p. 76–80), and thereby, it is highly functional for the encounter that will follow.

Together, these different elements of coordination show the influence of the three levels we introduced in §1: the individuals, the socio-cultural context and the social interaction. The interaction process, as we postulated at the beginning of this section, is to an extent autonomous in the unfolding of the greeting: no matter what kinds of coordinations are at play, none of them alone or their sum, or the individual mechanisms that aid in sustaining the interaction, can fully predict the actions that will occur, or the significances they will have for the interactors.

## 3. The interplays between interactional and individual autonomy in the co-creation of meaningful action

For interactional sociology, meaningful action can already be found at the level of the organization of interaction. Meanings of actions are conceptualized as *social* meanings, ordinarily as something that the interaction participants share; in a sense, as something that does not reside in the individual actors, but in the actions themselves. As seen from this perspective, actions are meaningful, rather than something that is endowed with meaning [38].

For enaction, however, a fundamental element is missing before we can speak of meaning: the individual person. In the enactive perspective, meaning is generated between persons participating in interactions—where both the individuals *and* the interactions are of equal importance. Persons are moving, animate, experiencing, social subjects. Enaction understands them too in terms of autonomy, as we will see below. It is time to bring these individuals into the fold. In this section, we shine a light on the interplay between subjects and the interactions in which they participate. Interactional sociology has interesting things to offer there too—perhaps unexpectedly, since it does not like to thematize the individual much—as we will see.

The interactional sociology perspective on the relationship between individual and interaction was encapsulated in Goffman's ([39], p. 3) famous phrase: ‘Not, then, men and their moments. Rather moments and their men’. In exploring the relationships between the individual and the interaction order, Goffman thus prioritized, at least methodologically, the latter. While he maintained that understanding the individual is necessary for the study of interaction, such understanding involves a psychology of a particular kind: ‘one stripped and cramped to suit the sociological study of conversation, track meets, banquets, jury trials, and street loitering’ ([39], p. 2). Below, we will argue that Goffman's psychology may in fact be more sophisticated than he wanted his readers to think. However, the position of enactivism is different. Enactivism not only emphasizes the autonomy of interaction, but likewise, the autonomy of the individuals participating in the interaction.

The enactive definition of social interaction requires that both the individuals involved *and* the interaction are autonomous, before we can speak of a social interaction. According to this definition, we can speak of a social interaction only if both the following conditions are met: (i) there is a co-regulation at the level of interaction dynamics that takes on an autonomous organization; and (ii) the autonomy of the individuals participating in the interaction is not destroyed in the process (though it can be de- or increased) ([7], p. 493). Enaction in this way makes explicit a necessary tension between individual participants on the one hand, and the interaction as a process on the other. If there is only interactional organization, we cannot yet speak of a *social* interaction. Similarly, if one of the participants completely dominates the interaction, we are not dealing with a social *interaction* (it would be like interacting with an object, not with another subject). Imagine a couple dance: one cannot lead unless the other assumes the role of follower, and if one participant does not contribute to the moves, it would be like carrying a doll across the dance floor. Thus, not only is the interaction process autonomous in terms of its internal organization, it also depends, crucially, on the autonomy of the individuals participating in it. In this way, for enaction, interactional organization requires both interactional and individual autonomy.

If both interaction and individual are autonomous systems, then they are in continual tension with each other in each ongoing interaction. These tensions get manifested in what might be called vulnerability. What is interesting about the confluence of enaction and interactional sociology that we propose in this paper, is that both the individual and the interaction can be conceptualized as vulnerable. Vulnerability hangs together closely with autonomy. It is at the interplay between individual and interactional autonomy and vulnerabilities that the co-creation of significance and significant action happens, as we will now show.

Let us first consider individual vulnerability. Vulnerability is intimately related to individual autonomy. Autonomy is at the basis of how an individual understands their world. Individual self-organization, which is to the deepest level embodied, naturally entails needs and constraints, since certain things a sense-maker encounters will be beneficial for its self-maintenance, and others will be pernicious. The first kind should be sought out, and the second avoided [5,10]. This is the intrinsic teleology of living self-organization, according to enaction.^4^

Living self-organizing systems are precarious, which means that they can be threatened (and, in the reality of the biological, physical world, *are* continually threatened, also from within—see the definition of precariousness and an explanation of its importance in the enactive framework [11]). When a self-organizing system has some way of anticipating or foreseeing such threats and adapting its actions and action tendencies to this, it can be said to be a sense-maker, for whom things have intrinsic, existential, affective, experienced significance [10]. Thus, vulnerability is at the heart of the very sense-making that characterizes living self-organizing systems, such as human individuals.

In spite of him claiming that his psychology is ‘stripped and cramped’, Goffman's work shows how particular vulnerability is at the heart of the individual's participation in social interaction. This vulnerability involves the self. Goffman [1] pointed out that an individual invests much psychic energy in their socially recognized self-image, or *face*. The face consists of positive social attributes. Therefore, it is essentially derived from the supplies that society can offer. Importantly, the individual face is also utterly dependent on interactional recognition and confirmation: our images as competent human actors, as men or women, or as incumbents of any other social identity are in the hands of our interaction partners [41]. Physical and spatio-temporal arrangements are also involved: claims for personal space and temporary occupation of objects such as chairs, objects attached to the body, rights to control others' access into conversation, as well as ownership of personal information [37]. Through violations of all this, the individual's self, or face, can be questioned. Hence, as Rawls ([42], p. 140) puts it, the ‘individual is never secure in an encounter’. In other words, the maintenance of face is a key aspect of individual autonomy. This aspect of individual autonomy is in constant need of ratification that can only be received by acting within the system of interaction.

Interactions as autonomous systems, too, are inherently vulnerable, misunderstanding being the most obvious and continuous threat for them. Thus, there are many different kinds of processes and mechanisms to secure mutual understanding in the face of potentially difficult circumstances. For example, to enhance language comprehension, participants have been found to draw on their immediately preceding experience to fine-tune their expectations about the kinds of phonetic and syntactic features of speech signal that are likely to occur in a given context [43,44]. The practices of conversational repair [4,45], then again, have evolved to deal with those moments of interaction when the threat of misunderstanding has momentarily realized; these practices have been argued to be of vital importance in securing and restoring intersubjective understanding in conversational interaction [46]. The operation of turn-taking also has its ruptures, for example, in simultaneous onset of two turns at talk or by one participant interrupting the other's turn. These ruptures can be in the service of activities at hand or can go against them, and they have standardized locally managed solutions [3,47,48].

A more subtle and yet most intriguing vulnerability of interaction has to do with engagement in encounters. In §2, we referred to the duality in Goffman's [39] account of engagement: while spontaneous involvement in conversation—something Goffman calls *unio mystico*—is both a highly valued experience and a social norm, the reality of an encounter is usually different, as the participants drift towards disengagement. External pre-occupations, as well as the participant's consciousness of their own performance, or that of the other, or concern about the unfolding of the interaction, regularly hamper the *unio mystico*. The social system of encounter is thus inherently ambiguous, as ‘spontaneous “normal” involvement seems to be the exception and alienation of some kind the statistical rule’ ([39], p. 134). But whether interactions are always vulnerable in this way, is a matter of empirical research.

Alongside his theory of face, Goffman's account of alienation is one of the places where he seems to have been concerned with the subjective experience of individuals. While downplaying the relevance of his own psychology, Goffman eventually proffered most insightful observations of the experience of interaction. But this was done, as it were, between the lines. For enaction, on the other hand, subjectivity and experience are from the beginning full and basic aspects of social interaction [49,50]. The felt efforts of maintaining self-organization are an integral part of enactive cognition. There is no meaning-making without a subject *to* or *for whom* things affectively and experientially make sense, even if it is not always explicit. Many preliminaries of making sense happen below, before, or on the cusp of awareness. In relation to social sense-making, we may even consider that there is often an ‘invisible excess of sense’, a presence of hidden, ineffable or even secret meanings, which is best left to play its role as precisely silent [51].

We can thus speak of a primordial tension between individual and interactive autonomy [25]. Individuals are almost continually engaged in different ongoing social interactions that influence them. Even when no other is immediately present, we engage in relational patterns that affect our sense-making and are affected by it, such that a social interaction is sustained over time. This is illustrated, for instance, in research showing how spouses' quality of life remains interdependent, even after one partner dies [52]. Balancing interactional and individual autonomy and vulnerability or precariousness, is therefore a matter of co-regulating the interaction, and of regulating one's participation in interaction [25,53].

One conceptualization of such co-regulation of interaction, relevant to enactivism and interactional sociology alike, comes from the dynamic systems theory of Beebe & Lachman [54]. Investigating mother–baby interaction and psychotherapy, they propose that there is an ‘intimate connection between self- and interactive regulation’ ([54], p. 22), as behaviours employed in self-regulation have equally a role ‘in influencing, and being influenced by, the partner’ ([54], p. 22). The linkage between self- and interactive regulation is bidirectional—that is, the means for regulating interaction also serve as means for self-regulation. Peräkylä *et al*. [55] recently applied this co-regulation perspective by investigating autonomic nervous system responses in tellers and recipients of conversational stories. The verbal and non-verbal displays of affiliation by the story recipient decrease the storyteller's level of arousal but increase that of the recipient. Affiliative behaviours (e.g. facial expressions, affective response tokens, verbal assessments) thus not only influence the course of the overt interaction, but also influence both participants' internal state. In this way, managing interaction and self-regulation in interaction is always a co-regulation.

Co-creation of meaning, then, happens when interactors *participate in each other's sense-making* [7], i.e. when interactive acts ‘achieve more than I intend to. And conversely, I can achieve what I individually intend to with less, through coordinated completion of the act by the other’ ([25], p. 13). Managing the tensions between interactional and individual autonomy, or dealing with breakdowns and transitions in coordination, can be done in different ways, and is at the origin of different forms of social agency and ‘participation genres’ (an extension of Bakhtin's ‘speech genres’), and ultimately of languaging behaviour. What kind of body the participants have, plays a basic role in this interactive management, and bodies have and develop particular sensitivities to and abilities in participation genres, to such an extent that we can even speak of ‘linguistic bodies' [25].

Let us once more return to our example of greetings. Research has shown that people have a preference for doing together, even if there are inherent tensions to it. In collaborating smoothly in the reaffirmation of their relationship, the participants most efficiently satisfy each other's face needs ([56], p. 390). Vulnerabilities are present, however. Greeting somebody who is not prepared to greet is a major threat of face [36], as is the choice of salutation that implies more or less relational intimacy or status difference than the other is prepared to show. Individual vulnerabilities and sensitivities correspond to the vulnerability of interaction: miscoordination of body movements and gestures, as well as the participants' behavioural trajectories momentarily depart. But eventually, the participants will find ways to participate in and generate the greeting. Even in the most routine situations, this involves co-regulation, as participants attend and respond to each other's actions, and thereby jointly shape the trajectory of the interaction and re-affirm or redefine their relationship.

## 4. Some interactive guidelines for studying the co-creation of meaning

In this paper, we have brought together enaction and interactional sociology to gain a fuller understanding of the co-creation of meaningful action. We would like to conclude on two points: (i) what both approaches can learn from each other, and (ii) implications for empirical research.

The enactive approach complements interactive sociology in showing that the individual can have a place in the conceptualization of interaction, without compromising the idea of the autonomy of social interaction. In this view, both interaction and individual can be conceptualized as autonomous systems, and it is possible to investigate the linkages between them. This reconceptualization gives a new theoretical significance to the classical Goffmanian observations on face and alienation from interaction. Viewing these in an enactive light encourages interactional sociologists to explore the subjectivity and experience involved in them. Of equal importance for interactional sociology is enaction's specification of different interlocking orders of temporality that exist in interaction. While interactional sociology (especially conversation analysis) has paid much attention to sequentiality, enaction encourages interaction sociologists to attend more to temporal complexity, offering analytical tools to also deal with phenomena such as synchronicity.

In turn, Goffman's work on face-work and alienation expands enaction's theoretical proposals about the interplay between individual and interactional autonomy. In the light of the so-called interactive turn in cognitive science [57] or, perhaps better, the intersubjective turn [58], interactional sociology may help further specify which events, structures, processes and properties contribute to the autonomous self-organization of the interaction process and how; and how they are therefore possibly in tension with the socio-cultural and individual levels. This should help generate and formulate hypotheses for empirical research. For instance, enaction has treated co-presence so far as a non-socially interactive situation (based on the enactive definition of social interaction). But enactive explanations of dispositions or ‘readiness to interact’ [59] may benefit from thinking, with interactive sociologists, about the question of what indeed happens when agents are co-present and aware of each other. Finally, interactional sociology's emphasis on structures is a demand for enaction to further clarify questions about the stability of modes of interaction, the settling of conventions, etc.

Our main message is that social interactions are autonomous, and as such can and should be studied in their own right to help answer the question of how we co-create meaningful actions. The next question is: how? What are the implications for the empirical study of social understanding?

In its most radical form, the idea that social interactions are fundamental for understanding the co-creation of meaning has led to proposals such as the interactive brain hypothesis, according to which interactive experience and skills form the basis of social understanding and of social brain functioning [59,60]. As we explained in this paper and elsewhere [61], this does not mean to just turn the individualistic logic of psychology and neuroscience on its head, and to focus only on interaction. Instead, it is necessary to understand both the interactive *and* the individual contributions to the (co-)regulation and coordination of behaviours together [62].

Coordination in social interactions is robust, and at the same time it has both structural and emergent aspects, which are in continual interplay and tension with each other, as well as with the processes of individual self-maintenance. Using the taxonomy of structures and processes that contribute to the self-maintenance of social interactions (introduced in §2) will allow empirical research on the different aspects of coordination. For example, given the fundamental significance of turn-taking and repair organization for human joint endeavours, one line of current research involves searching for variability and universality in the turn-taking structures [17,20] (for an extension of this line of research on marmosests, see [63]) and in practices of repair [64] across languages and cultures. Furthermore, sequentiality, as a basic form of coordination of interaction, rests upon the participants' ability to recognize each other's behaviours as specific actions that call for specific responses. Recent studies by Gisladottir *et al*. [65,66] have started to unravel the temporal organization and the neural underpinnings of conversational action recognition processes. In addition, given the fine nuances of coordination associated with the ‘mere’ spatio-temporal presence of other persons, studies on the neural and experiential responses to such events would be motivated ([60], p. 186, [67]). Finally, considering the rich experimental research tradition on the ways in which individuals respond to threats to their self-image (e.g. [68,69]), Goffmanian theory of the interactional maintenance of face can inform experimental designs in this field. This would enrich our understanding of the behavioural, experiential and neural aspects of the basic interactive vulnerability of self.

As pointed out above, what contributes to the self-organization of interaction can have different origins, ranging from the rules of turn-taking to shared interactional histories and pre-coordinations based in socio-cultural customs and practices. For example, experimental subjects' neural activity can synchronize when they view the same emotional episode of a film or hear the same story [70,71]. Based on the perspective that we have we presented, we can understand this as external coordination: it is a neural synchrony on the basis of coupling to something in common. Understanding other origins or references of interactive coordination—particularly in terms of coordination-with—however, calls for further research efforts.

For this kind of work, dynamical systems techniques can be useful. In line with our general point, these techniques can reveal aspects of the deep structure of social interactions as organized systems. They can indicate the presence of interaction-dominant dynamics (i.e. situations where the system components cannot be said to function as independent units but show activity correlation across many timescales) [72]. Dynamical systems can differentiate between different references and origins of coordination. For instance, they can distinguish leaders and followers [73,74], and other factors affecting coordination. Dynamical systems could also be used to study the interplay between self-regulatory behaviour (such as breathing, heart rate, etc.) and interaction-regulation (e.g. [75]). Or they may be used to assess which factors of alienation, as Goffman called them [39], are at play. We would need to determine how to measure the presence of individual pre-occupations, which could be done with eye-tracking to follow participants' attention, or by studying body movements of the participants in relation to each other (e.g. through proxemics research [76]). More subtle dynamical signatures of whether participants are ‘in the flow’ of the shared situation can be probably also be found. Movement trajectories may be different when participants are very engaged with each other, versus when they are more busy with self-regulation, when interaction breaks down, when it is being repaired, and so on. The measure of correlations between processes happening at different timescales can be used to distinguish the presence of skillful flow [77]; similar techniques could be adapted to differentiate between levels of interactive versus individual engagement. Finally, dynamical systems tools may also be used to examine where which kinds of perturbations have an impact, and on what (on interaction or on participants, on which structures, processes or behaviours, etc.) [78,79].

Clearly, the full range between laboratory-controlled and naturally occurring situations should be studied. It will be important to keep in mind that our own recognition of another person's meaningful actions depends on what we are currently doing, on the local relationships of contingency that emerge between our behaviours, as well as on the normative expectations governing the specific types of sequences and activities that we are engaged in. Moreover, all this is intertwined with our individual and joint interactional histories. We would therefore like to encourage novel approaches that address several of these aspects at once—something that is already increasingly possible.

## Endnotes

1 In this paper, we use the terms ‘action’, ‘meaning’ and ‘meaningful action’ interchangeably, to refer to our main issue. Our concern here is not definitional issues around the terms action, meaning or meaningful action, but rather to contribute to a better understanding of how people do things together that make sense to them in one way or another.

2 The enumeration here does not reflect any kind of hierarchy or order, it is just here to clarify which are the levels at issue.

3 We are not really sure what would be the largest timescale, which is why we leave it open-ended.

4 And this is one way in which autonomy of individuals and interactions are different. For lack of space, we cannot go into further differences here, but see, e.g. [40] for specifications of individual agency.
